# The AIDS Epidemic and Its Control

**Published:** 1987-08

**Authors:** John Seale

**Affiliations:** Late Consultant Physician, Departments of Genito-urinary Medicine and Venereology, St Thomas's Hospital, London


Bristol Medico-Chirurgical Journal Volume 102 (iii) August 1987

### The Aids Epidemic and its Control

**John Seale MD, MRCP**
Late Consultant Physician, Departments of Genito-urinary Medicine and Venereology, St Thomas's Hospital, London.

### INTRODUCTION

No rational decisions can be made about the control of AIDS without a clear understanding of the nature and severity of the epidemic, the means of transmission of the virus and the prospects of cure or preventive vaccine. The key scientific facts underlying the epidemic are quite simple. Once the public is fully informed of the true nature of the threat that faces us all, it will accept the measures essential to halt the spread of the virus, even though they will inevitably require curtailment of the liberty and civil rights of everybody—as in war-time.

### THE NATURE OF THE DISEASE

AIDS is a contagious, infectious, communicable disease caused by a lentivirus (slow virus), a member of the family of retroviruses. No lentivirus has been known to affect humans before the advent of AIDS. AIDS has a prolonged, silent incubation period of great variability, usually lasting several years, followed by slowly progressive disease always ending in death. An epidemic of such a disease is the ultimate virological nightmare.

### MORTALITY FOLLOWING INFECTION

Within 5 years of infection with the virus, 25% of people have developed full blown AIDS and all of them die. The ultimate mortality within 20 years of infection is unknown as the virus has been spreading for only 10 years. Many virologists now accept the pessimistic view that all people infected with the virus will eventually be killed by it. All virologists are agreed that once infected with the AIDS virus, people are potentially infectious to others for life.

### FAILURE OF ANTIBODIES OR VACCINES TO PROTECT

In contrast to most virus infections, antibodies to a lentivirus do not provide immunity, they fail to neutralise or eliminate it. Although many people infected with the virus look and feel well for several years, destruction of the brain and immune system is progressing slowly. The outlook for a successful vaccine is bleak. None is available for the lentivirus diseases of animals. Search for a vaccine against infectious anaemia of horses for eighty years and against maedi-visna in sheep for forty years, has proved futile. Indeed when antibodies to a lentivirus are produced artificially by vaccination, vaccinated animals die after subsequent infection more rapidly than those which have not been vaccinated. In spite of a number of successful vaccines that have been produced, it should be realised that for the majority of viral and bacterial diseases vaccines do not work.

### BLEAK OUTLOOK FOR A CURE

No simple, effective, curative drug like penicillin, will be available for AIDS in the foreseeable future, because once a person is infected, the viral genetic code is permanently inserted into the human genetic code of cells in the brain and other tissues. Any drug, such as AZT, which blocks replication of the virus, thereby halting progress of the disease, will have to be taken continuously for life. All drugs used so far are highly toxic and expensive. If a cheap, apparently effective drug became available it will take several decades to be certain that it is both effective and safe. Nevertheless, many companies will announce 'promising' new drugs and 'breakthroughs' in the treatment of AIDS for simple commercial motives. The handling of the recent AZT clinical trials by the US Government was particularly important. The US Public Health Service insisted the trials cease long before any long term benefit of the drug had been shown and before the manufacturing company suggested it, thereby misleading the public into believing that a 'cure' for AIDS was already in the pipeline. Such disinformation weakens the political will to implement the control measures required to halt the spread of the virus.

### TRANSMISSION OF AIDS—SEXUAL INTERCOURSE

Scientists and doctors have repeatedly stated as fact that the AIDS virus is fundamentally transmitted during sexual intercourse, but is unfortunately sometimes transmitted in blood. This is highly misleading. In reality AIDS is characteristically a blood borne infection, which is only transmitted with difficulty during sexual intercourse. The illusion that AIDS is essentially a sexually transmitted disease arose from the first observation that AIDS appeared to affect only sodomites with numerous partners. However sodomy is not sexual intercourse in the biological sense of the word. Homosexual men engaged in homosexual activities frequently insert their fingers, fist, penis or tongue into the lower intestinal tract of their partners. These manoeuvres transmit any virus which persists in the blood for months or years with devastating efficiency, even though no virus is present in semen or saliva. This has been shown very clearly with hepatitis B virus which, in prosperous communities, infects the majority of homosexual men within three years of their becoming sexually active, whereas hepatitis B virus infection remains rare amongst heterosexual men and women, even though they frequently change partners.

### DISINFORMATION FROM SCIENTISTS

The AIDS virus persists in an infectious state (ie as cell-free virions) in blood and serum at levels up to 25,000 virions per ml. according to the only published paper giving this critically important information. Cell free virions were detected easily in saliva over two years ago, but quantative studies have still not been published. No infectious virion has been detected in semen according to the only two detailed published studies on the subject, which between them included a grand total of only 3 men examined. Virions have been detected in the vaginal secretions in only trivial quantities.

The scale of the deceptions and misinformation perpetrated by virologists, clinicians and editors of scientific and medical journals about the infectivity of genital secretions, compared with that of blood, serum and saliva, has been astonishing. A negligible amount of research has gone into the critical matter of transmission. A few preliminary papers were published and their findings repeatedly quoted as showing the opposite of what they actually showed. When this was pointed out in letters to the editors of major medical and scientific journals, publication has been refused. No attempt has been made to check, double check and recheck the findings in other laboratories and in other countries, or to rectify published errors.

As far as it goes, the tiny research effort into infectivity of body fluids indicates that saliva is more infectious than genital secretions, but that blood and serum is vastly more infectious than either. Consequently the idea that condoms can have any significant effect on the spread of AIDS in a nation is utterly preposterous. Governments all over the world are spending millions of pounds advising their citizens to prevent AIDS by using condoms on the basis of manifestly fraudulent misrepresentation of scientific evidence presented by scientists themselves.

The AIDS virus is unusually stable outside the human body. It retains almost all its infectivity after seven days in water at room temperature and some after being kept dry for a week. A virus with this degree of stability, which persists in the blood and is shed in saliva, cannot possibly fail to be transmitted in many ways apart from sexual intercourse.

### VARIABLE 'EFFICIENCY' IN MEANS OF TRANSMISSION

A virus which persists in moderate quantities in the blood for years and is shed in small quantities in saliva will be transmitted with far greater ease by some means than by others. Injection of the virus through the skin in hypodermic needles is the most certain method of transmission, as is common amongst drug addicts in the West and in third world hospitals where sterilisation of needles is defective. It also occurs when virus-contaminated blood transfusions and clotting factors are administered.

Male homosexual contact of the finger, penis or tongue with the rectal wall of another man transmits the virus very easily. 70% of the male homosexual population of San Francisco were infected within six years of the arrival of the virus in the city, and nearly 30% of London homosexuals are already infected. The percentages are rising remorselessly in large cities throughout the Western world, unaffacted by the highly acclaimed 'safe sex' propaganda. Well over 50% of new-born babies of infected mothers are infected.

'Moderately efficient' means of transmission include mouth to mouth and genital contact before and during normal sexual intercourse, oral salivary contact between small children, needle stick injuries to nursing staff, and chance contact of sores and abrasions with blood, serum, saliva or sputum.

'Inefficient' means of transmission include social kissing, inhalations of respiratory aereosols caused by coughing and sneezing and blood sucking insects.

Transmission by inhalation is only 'inefficient' because of the relatively small number of virions in bronchial secretions. However if an AIDS virion is inhaled into the lung it is engulfed by an amoeba-like macrophage within which the virus can replicate and initiate infection. AIDS patients with the complication and pneumonitis suffer from a chronic cough and produce aerosols containing the AIDS virus, minute dry flakes persist and float in the air indefinitely. The maedi-visna lentivirus infection of sheep is a respiratory infection and is transmitted by respiratory aerosols when they are crowded together in winter shelters. It is not sexually transmitted.

Transmission of AIDS by biting insects will depend upon the quantity of virions in the blood of the bitten person, the number of such people around, and the anatomy and feeding habits of the biting insect. Infectious anaemia of horses, a lentivirus disease of horses is characteristically transmitted by large biting insects, particularly stable flies and horse flies. It is not sexually transmitted. The AIDS virus has been shown to remain infectious in the stomach of bed bugs for at least two hours. It has been shown that it can infect other insects, including mosquitos and cockroaches. Replication of the virus in insect cells has not yet been demonstrated.

### SATURATIUON OF THE BRITISH POPULATION WITH THE AIDS VIRUS

The basic facts are that the entire population is susceptible to infection and that once infected they remain potentially infectious to others for life. As the number of people infected rises the probability of transmission by any particular contact also rises. Initially the virus was introduced into Britain from the USA by homosexual men by having frequent 'efficient contacts'—sodomy with strangers. As the number of infected homosexuals rises so does the chance of being infected by a single contact until the homosexual population is saturated. Similar 'efficient contact' has spread the virus rapidly among drug addicts. As the number of infected homosexuals and addicts has increased so the number of people infected by receiving a blood transfusion or clotting factor has increased.

Once a critical mass of infected people has been created by 'highly efficient' contacts, then 'contacts' which are only moderatly efficient but occur very frequently, such as normal sexual intercourse or small children playing together will spread the virus in ever widening circles throughout the population. Finally, 'highly inefficient contacts' which occur very frequently indeed, such as coughing and sneezing in public and being bitten by insects, will infect many people as millions of infected persons interact with the non-infected, and saturation of the entire British population becomes unstoppable.

### MOTIVES FOR THE MISINFORMATION OF THE PUBLIC

Homosexual men have been the most determined and effective group in distorting the truth about AIDS. They have been so effective because there is a scattering of homosexuals amongst all key professional groups involved—scientists, doctors, medical editors, journalists, lawyers, politicians and priests. The initial impact of AIDS on homosexuals in the West inevitably resulted in an unusually high proportion of them becoming involved with the disease since it first surfaced. Many of the men who are particularly knowledgeable about and dedicated to AIDS research, treatment, legislation, publication and education are homosexuals. Most in the professions are only identifiable as homosexuals to other men with similar inclinations—few have 'come out' and even the wives of those who are married are usually unaware of their habits. Hence they automatically form a type of secret society without even trying, with wide ramifications across professional, institutional and national boundaries.

Homosexual men have been the main vectors of the virus throughout the Western world and if it had not been for their activities very few people in prosperous countries would be infected. Many do not wish to face reality because of guilt, most do not wish to change their ways, and a few seeing death and destruction facing themselves and their friends are dedicated to destroying the rest of society with them. All wish to deny the reality that restricting the freedom of homosexuals to infect each other and other people is essential if our society is not to be destroyed by the virus.

For a variety of reasons that have been touched on above, misinformation has come from scientists and scientific journalists, from doctors and from politicians.

### VARIETIES OF MISINFORMATION

People with AIDS are characterised as belonging to a small 'risk group' giving the false impression that the vast majority of people cannot get AIDS. AIDS is portrayed as only a behavioural disease caused by narcotic and sexual misdemeanours. This implies that if anybody gets AIDS it is their own fault. Emphasis on transmission of the virus during sexual intercourse, and education as a solution to the epidemic implies that the disease will disappear with modifed behaviour. This missed the point that as the epidemic explodes, infection by chance, non-sexual contact becomes ever more common. By equating sodomy with sexual intercourse the impression is given that homosexuals have just been unlucky to be infected before heterosexuals. In reality sodomy has spread the virus through the population at a vastly greater speed than normal sexual intercourse could ever achieve.

The value of blood tests for diagnosis of AIDS virus infection is repeatedly denigrated by those who do not want them introduced compulsorily. In fact the blood test is an unusually reliable diagnostic tool. The suffering of those with AIDS is highlighted while the suffering of those who will get AIDS in future if appropriate steps are not taken is ignored. The rights of those infected with the virus are stressed, while the rights of the uninfected to be protected from a lethal virus are ignored. Protection of the safety of its citizens is one of the major obligations of the State.

### METHODS OF CONTROL

The most urgent step to be taken is to break the pervasive grip of homosexuals on the information and disinformation which has emanated for so long from the journals of science and medicine and the media. Speed is of the essence because every day that is lost will increase the human misery which, in any event, will be vast. We are facing a national catastrophe equal to any in the history of the nation. The life of every citizen is at stake. Death from AIDS is a protracted horror unequalled by other disease. *The only way to halt the spread of the virus is to identify all those who are infected by compulsory testing. Government must then take whatever steps are required to ensure that those infected do not pass the virus on to anybody else.* The longer this action is delayed the greater will be the task when it is finally undertaken, and the greater the danger that the spread of the virus will then be unstoppable. The actions required by Government are comparable to those that need to be taken in waging a war of survival.

[Paper given to the Bristol Medico-Chirurgical Society Feb 18th, 1987, based on a memorandum to a Select Committee of the House of Commons.]

